# Androgen Receptor as a Potential Target for Treatment of Breast Cancer

**DOI:** 10.16966/2381-3318.129

**Published:** 2016-10-06

**Authors:** Y Wu, JV Vadgama

**Affiliations:** 1 Department of Medicine, Division of Cancer Research and Training, Charles R. Drew University of Medicine and Science, Los Angeles, California, USA; 2 Jonsson Comprehensive Cancer Center, David Geffen School of Medicine, University of California, Los Angeles, California, USA

**Keywords:** Androgen receptor, Breast cancer, Estrogen receptor alpha

## Abstract

Clinical studies have shown that the androgen receptor (AR) is ubiquitously expressed in breast cancers and this could provide prognostic implication in the diagnosis and treatment of breast cancers. Data from Nurse’s Health Study on women with invasive breast cancer suggest that a significant number of tumors were AR-positive as defined by immunohistochemistry. In addition, the distribution of AR among different breast cancer subtypes varies significantly, and the biological reasons for this variation are not well understood. Despite strong histochemical evidence, the AR status is not applied for assessing pathological findings and disease outcome in clinical practice. AR antagonists are not currently used as therapy in breast cancer. This is in part due to conflicting results from early clinical trials with first generation of AR antagonists together with the complexity in breast cancer heterogeneity. In addition, role of AR in breast cancer is not fully understood. Here we will review the role of AR in different subtypes of breast cancers and elucidate its mechanisms. We will also discuss some recent interesting findings on the second generation of AR antagonists for treatment of breast cancer.

## Introduction

Besides estrogen and estrogen receptor (ERα), there is emerging evidence that demonstrates the importance of androgen and androgen receptor (AR) in breast cancers [1]. It is well known that androgen can influence breast cancer cell growth by either aromatization to estrogens or through AR-dependent mechanisms. Epidemiologic findings also demonstrated an association between circulating levels of androgens/testosterone and the risk of breast cancer in both premenopausal and postmenopausal women after adjusting for estrogen levels [2–9]. At cellular level, co-localization of AR and ERα in breast cancer tissues [10] implies that the nuclear interaction of AR and ERα signaling could also influence breast cancer cell growth. However, AR signaling can operate without interacting with ERα at cellular level since many AR-positive/ERα-negative cells exist in different subtypes of breast cancer [10]. Hence it is not surprising that the clinical prognostic role of AR in primary breast cancer varies in different subtypes of breast cancers. Hence it is critical to better understand biological functions of AR in breast cancer and appropriately select patients for AR targeting therapies.

## AR Expression in Breast Cancer

ERα-positive breast cancer accounts for 75% to 80% of breast cancers and it determines the use of antiestrogen (tamoxifen) or aromatase inhibitors in the adjuvant treatment. AR is also expressed in 74% to 77% of breast cancer cases [11,12]. The prevalence of AR expression in ERα-positive breast cancer ranged from 60% to 80% and in ERα-negative was 10% to 50% [12–14]. A subtype of breast cancer is negative for ERα, but has enriched HER2 (epidermal growth factor receptor 2). The expression of AR was found in 50% to 60% of ERα-negative/HER2-positive breast cancer [12]. Another type of breast cancer is negative for the ERα, progesterone receptor (PR) and HER2 called triple negative breast cancer (TNBC). The AR is found to be positive in 10% to 32% of TNBC [15]. AR-positive breast cancer is also more likely to be associated with PIK3CA mutation, but has no association with P53 mutation [16,17].

## Prognostic Discrepancy of AR in Breast Cancer

AR expression appears to play different prognostic role in ERα-positive and ERα-negative breast cancers. In ERα-positive breast cancer, AR was reported to predict favorable disease outcome consistently [16,18–20]. Co-expressing AR in ERα-positive breast cancer improved disease-free survival (DFS) and overall survival (OS) significantly [18–20]. The AR could serve as an independent prognostic factor for DFS of ERα-positive breast cancer [18]. The mechanisms may involve AR-dependent androgen signaling pathway controlling estrogen-induced cell proliferation [21]. Furthermore AR could repress ERα activity by competing with ERα for binding to regulatory regions of ERα target genes [10]. Recent studies suggest that activation of AR by androgen directly stimulates the expression of tumor suppressor *PTEN and KLLN*, a newly identified tumor suppressor gene which shares a bidirectional promoter with PTEN, resulting in cell growth inhibition and transcriptionally activation of p53/p73-mediated apoptosis in breast cancer [22,23].

The prognostic role of AR in ERα-negative breast cancer is debatable. AR-positive status was associated with a significant better DFS in TNBC in some studies [14,24], but it was correlated with poor disease outcome in other studies [19,25]. The study that reported AR predicting better DFS in ERα-positive tumor showed no impact on survival of TNBC by AR, but having trends of poorer outcome in the ERα-negative with HER2-positive status only [18]. Besides data from a systematic review and meta-analysis included nineteen studies with a total of 7693 women showed that expression of AR in women with breast cancer was associated with better overall survival (OS) and DFS irrespective of ERα status [20]. However the limitation of meta-analysis of the literature included heterogeneity of resources in patient population and lack of standardized methods of assessing AR. The prognostic discrepancy of AR in ERα-negative breast cancer may be also due to the differential molecular features of the ERα-negative tumors and the lower frequency of ERα-negative compared to ERα-positive breast cancers. Further assessment of the prognostic role of AR in ERα-negative breast cancer with well-designed clinical studies is required.

Although the importance of AR in breast cancer has been challenged, there is sufficient evidence that its action in ERα-negative breast cancer may involve increase in cell proliferation and anti-apoptosis, steps that lead to poor outcomes from cancer treatment. These mechanisms may be associated with activation of the receptor tyrosine kinase signaling pathways. It has been shown that the AR-positive type of TNBC is more likely to have activation of EGFR (Epidermal growth factor receptor) and PDGFRβ (platelet-derived growth factor receptor), and have enriched for *PI3KCA* activating mutations [26,27]. Dual targeting AR pathway and EGFR and/or PDGFRβ signaling, or AR and PI3K/mTOR pathways additively inhibited cell growth and cell viability [26,27]. Similarly dual targeting AR and ERK1/2 pathways also observed an additive anti-proliferative effect in TNBC cells [28].

AR activation has also been linked to transcriptional induction of Wnt7B and activation of Wnt/β-catenin pathway in the ERα-negative/HER2-positive breast cancer cells [29]. The AR is able to induce HER2/HER3 dimerization through FOXA1 (Forkhead box protein A1) in the HER2 enriched ERα-negative breast cancer cells [29]. These data suggests the role of AR in HER2 enriched ERα-negative cells could lead to resistance to trastuzumab since both Wnt/β-catenin pathway and HER2/HER3 dimerization are associated with trastuzumab resistance [30]. Recent studies demonstrate that targeting AR by anti-androgen drug enzalutamide synergistically improves anti-proliferation effect of trastuzumab [31].

## Targeting AR in Breast Cancer

Anti-androgenic therapies are well established for treating prostate cancer. Similarly, these strategies could be used cost-efficiently for the treatment of breast cancer. The results from an early trial using flutamide, a first-generation of antiandrogens, to treat metastatic breast cancer was disappointed due to “unselected” recruitment of patient population irrespective of their AR, ER, or PR status [32]. However, follow up studies with better understanding the AR action in different subtypes of breast cancer in well-designed clinical studies using next generation of antiandrogen therapy showed encouraging results in a selected group. For instance, a phase II trial with bicalutamide suggested potential benefits of targeting AR in AR-dependent, ER-independent breast cancer. A 19% clinical improvement was observed with bicalutamide over six months in a select group of patients with ER/PR-negative, AR-positive breast cancer [33]. Most recently the second-generation of non-steroidal antiandrogen, enzalutamide has been developed. Compared to bicalutamide, enzalutamide has approximately 5- to 8-fold higher binding affinity for AR and inhibits AR translocation to the cell nucleus [34]. AR plays a key role as a DNA-binding transcriptional factor that regulates gene expression in the cell nucleus. Hence, inhibition of AR nuclear translocation by enzalutamide could directly prevent transcriptional activation of AR-induced tumor associated genes that lead to cell proliferation and metastasis in breast cancer. In contrast to treatment with flutamide or bicalutamide, there has been no evidence of hepatotoxicity in response to enzalutamide treatment [35]. Recent studies using enzalutamide as a single agent in advanced TNBC patients has shown encouraging clinical outcome [26]. The 24 weeks clinical benefit rate was 42%–60% for enzalutamide in treating patients with stage II AR-positive TNBC [36]. Better safety, tolerability and pharmacokinetic profiles in well-defined patient population suggest significant clinical improvement with enzalutamide treatment. Since enzalutamide is able to inhibit AR nuclear activity, it should block AR-induced transcriptional activation of Wnt signaling. Subsequently, this action could prevent HER2-enriched ERα-negative breast cancer becoming resistance to trastuzumab [29,30]. Indeed an open-label trial to assess the efficacy and safety of enzalutamide with trastuzumab in subjects with HER2-positive/AR-positive metastatic or locally advanced breast cancer (ClinicalTrials.gov Identifier: NCT02091960) is underway currently. AR could repress ERα activity in ERα positive breast cancer by competing with ERα for binding to regulatory regions of ERα target genes and inhibit cell proliferation [10]. Hence, selecting patients with appropriate subtype of breast cancer for enzalutamide treatment could be very critical. The function of enzalutamide in ERα-positive breast cancer needs to further studies.

Current understanding of AR and AR signaling suggest potential for novel therapeutic targets for breast cancer. Many clinical studies are underway, such as a feasibility study of adjuvant enzalutamide for the treatment of early stage AR-positive triple negative breast cancer (clinicaltrials.gov: NCT02750358). A prospective single center Phase II study of bicalutamide as a treatment for AR-positive metastatic triple-negative breast cancer (mTNBC) patients (clinicaltrials.gov: NCT02348281) is currently open and actively recruiting participants [37]. The ongoing trials also include a cancer research UK phase I/II open label study to evaluate the safety, endocrine effects and anti-tumor activity of abiraterone acetate (CB7630) in patients with ER or AR positive advanced or metastatic breast carcinoma (clinicaltrials.gov: NCT00755885, CDR0000614059, CRUK-CR9304-21 and EUDACT-2007-003240-30). The results from those trials will provide more evidence and guidance on using antiandrogens for the treatment of breast cancer.

## Conclusion

In conclusion the detailed mechanisms of AR action in breast cancer, especially in ERα-negative breast cancer, still needs further elucidation for better assessing the clinical benefit of targeting AR therapies. The “AR-positive” needs to be well defined and the methods/antibody need to be standardized. To do so the role of AR in normal breast tissue needs to be fully elicited. Nonetheless it is clear that routinely assessing AR status at the time of diagnosis for breast cancer should be encouraged that will allow the AR status along with ERα/PR and HER2 status to be used for selecting therapeutic treatment for breast cancer. TNBC lacks specific treatment options, and AR positive TNBC patients have been reported to have poor pathological complete response to chemotherapy [36]. Therefore targeting AR and modulating its interactions with other signaling and factors could benefit AR-positive/ERα-negative breast cancer and improve disease-free survival.
